# Modelling and simulation of chemical reaction of porous MgCl2 pellets with NH3 by including impact of heat and mass transfer and structure change

**DOI:** 10.55730/1300-0527.3561

**Published:** 2023-04-07

**Authors:** Zeynep Karakaş HELVACI, Gürkan KARAKAŞ, Yusuf ULUDAĞ

**Affiliations:** Department of Chemical Engineering, Faculty of Engineering, Middle East Technical University, Ankara, Turkey

**Keywords:** Magnesium chloride, ammonia, modelling, transport phenomena, reaction kinetics, chemical heat pump

## Abstract

The MgCl_2_–NH_3_ reactive system is investigated in terms of heat and mass transfer coupled with chemical reaction through numerical simulation. The reversible nature of the chemical reaction is captured by including adsorption and desorption terms in the rate expression simultaneously. The kinetic coefficients of the adsorption are directly adopted from the literature, while those for the desorption reaction are calculated based on the thermodynamic relations. The impact of changing pressure and pellet porosity are also investigated in the simulations. The initial temperature of the pellet is 300 K in all simulations. Temperature, NH_3_ pressure, and conversion distributions in the pellets, along with pellet swelling are obtained and presented as a function of time. The results indicated strong effects of heat transfer resistances in the pellets.

## 1. Introduction

A considerable amount of industrial energy consumption is discharged to the environment as waste heat. Part of this waste energy can be recovered by upgrading the waste heat, which would improve the overall thermal efficiency of the process [1]. Heat pumps, then, have emerged as a useful method for waste heat recovery. On the other hand, the presence of a compressor and the use of hazardous refrigerants are the two shortcomings of conventional heat pumps. Chemical Heat Pumps (CHP) has emerged as an environmentally friendly alternative since upgrading and recovery of waste heat is achieved by a reversible solid-gas chemical reaction instead of the compression and decompression cycles in conventional heat pumps. In CHPs, environmentally benign refrigerants, e.g., water, ethanol, ammonia, and sorbents, e.g. zeolites, activated carbon, silica gel, and earth metal salts, are used. Furthermore, CHPs enable heat recovery or refrigeration over a wide range of temperatures based on the thermicity of the reversible reaction [2, 3]. Due to the high sorption capacities, the earth metal salts-ammonia have been a promising pair to employ in CHPs. For example, NH_3_ sorption capacity of 1 mole of MgCl_2_ is 6 moles. Low material cost, fast reaction rate, and convenient adsorption/desorption temperature ranges are other important advantages of using MgCl_2_-NH_3_ pair in CHP applications. However, very low thermal conductivities of the salts (0.1–0.5 W m^−1^ K^−1^) are the major problems during the heating and cooling cycles of the heat pumps [4, 5]. Although studies show that using a suitable binder (such as ENG, carbon fiber, etc.) increases the heat and mass transfer properties, the addition of binders is not preferable because of decreasing the sorption capacity of the salt. Another drawback of this system stems from the irreversible volume increase of the salt crystals after the first adsorption-desorption cycle since the molar volume of MgCl_2_.6NH_3_ is approximately two times higher than that of MgCl_2_.2NH_3_ [6]. This irreversible swell of the salt grains damages the structural integrity of the bed [7–10].

In order to overcome the specified major problems, metal salt-NH_3_ reactions have been investigated in many earlier theoretical and experimental studies. The “shrinking core model” and “particle/pellet model” are widely employed in the literature to capture the salt-NH_3_ reactions. Goetz and Marty [5] modeled the MnCl_2_-NH_3_ reactive system by using the grain-pellet model, in which the reaction in each grain was assumed to follow the shrinking core model. They kept the pellet porosity and size of the grains constant while allowing the pellet volume to change due to the reaction. They reported that the progress of the reaction zone was controlled by the conductive heat transfer. In the theoretical study of Lu et al. [11], both heat and mass transfer were considered at the pellet level, and it was assumed that there was no structural change. They reported that at low pressure (<1 bar), permeability was an important factor for the reaction of global advancement. Han et al. [12], also reported the importance of permeability for the MgCl_2_-NH_3_- graphite reactive system. Graphite was added to the reactive medium to enhance the system’s thermal conductivity and permeability. According to their results, heat transfer was not a limiting factor, while the system became mass transfer limited, especially at low pressures and high graphite amounts. Mofidi and Udell [6] also enhanced the thermal conductivity of the reactive system by adding graphite. They observed a heat transfer-limited system when the permeability was high. All these studies have shown that, as an alternative to time-consuming and costly experiments, mathematical modeling and simulation of metal salt-NH_3_ reactions can be instrumental in adopting them to some crucial applications such as NH_3_ storage or chemical heat pumps. In the earlier modeling studies, the metal salt-NH_3_ reaction kinetics was incorporated into the calculations by switching between adsorption and desorption expressions based on the reaction conditions. One of the first suggested kinetic model was reported by Mazet et al. [13]. They expressed reaction rate as the combination of reaction progression term and rate constant term for adsorption and desorption, individually. The reaction rate constant term was defined as the multiplication of Arrhenius term and pressure equilibrium drop term. The reaction conditions were set based on this pressure equilibrium drop term. For example, when the pressure was higher than the equilibrium pressure at the specified temperature, they used only the adsorption reaction rate expression while the pressure was lower than the equilibrium pressure then they switched to the desorption rate expression. Many studies were conducted on this topic and researchers mainly focused on the equilibrium drop term in terms of pressure drop term [4, 14], temperature drop terms [15], or the combination [16]. Still, the common point of all these studies is that reaction rates are given separately for adsorption and desorption reactions. On the other hand, the observed reaction is the outcome of these two simultaneously competing reactions, i.e. adsorption and desorption. Therefore, a reversible reaction kinetics model that includes both adsorption and desorption terms is potentially more accurate than those used in earlier studies in predicting net reaction rate at a given condition. In the presented study, theoretical reversible reaction rate expression for the MgCl_2_-NH_3_ reactive system is obtained by using literature values in the relevant thermodynamic relations. Also, the MgCl_2_-NH_3_ reactive system is modeled and simulated considering both heat and mass transfer and the reversible reaction. The operating parameters are selected to ensure net adsorption reaction. The particle-pellet model is used to investigate heat and mass transport at the pellet level. In addition, the volume change in the salt pellet during the reaction is considered and modeled to investigate its impact on the process. The effect of gas NH_3_ pressure change on the heat and mass transfer characteristics, conversion, and pellet volume change are simulated. The results are presented in the following sections.

## 1. Model development

### 2.1. Thermodynamic equilibrium of MgCl_2_-NH_3_

The generalized form of the reversible solid-gas chemical reaction taking place in a CHP can be expressed as given in Equation 1.


1 $$\text{S}\text{. }{\text{nNH}}_{3}+\Delta {\text{H}}_{r}↔{\text{S}}^{\prime }(\text{n}-\text{m}){\text{NH}}_{3}+{\text{mNH}}_{3,(\text{g})}$$

In Equation 1, S, and S^′^ represent metal salt, n, and m are the stoichiometric coefficients, and ΔH_r_ (J mol^−1^) is the heat of the chemical reaction. The reactive system between metal salt and ammonia is monovariant; hence the equilibrium pressure and temperature are related according to the Van’t Hoff equation for a reversible reaction given in Equation 2.


2 $$\text{ln }\left(\frac{{\text{P}}_{\text{eq}}}{{\text{P}}_{0}}\right)=-\frac{\Delta {\text{H}}_{\text{r}}}{{\text{RT}}_{\text{eq}}}+\frac{\Delta {\text{S}}_{\text{r}}}{\text{R}}$$

In Equation 2, P_eq_ is the equilibrium pressure in the MgCl_2_ - NH_3_ system, ΔS_r_ (J mol^−1^ K^−1^) is the entropy change of the reaction, R is the universal gas constant (8.314 J mol^−1^ K^−1^) and T_eq_ is the equilibrium temperature (K). The reaction between MgCl_2_-NH_3_ occurs when the temperature and/or pressure deviate from the equilibrium conditions.

### 2.2. Reaction rate expression

The reaction mechanism between metal salts and ammonia is reversible and complex due to the simultaneous adsorption and desorption reactions. The suggested rate expression by Mazet et al. [13] was modified, and the kinetic expression given in Equation 3 was used in the model to capture adsorption and desorption effects simultaneously.


3 $$\frac{\text{dx}}{\text{dt}}={\text{k}}_{\text{ads}}(1-\text{x})\frac{{\text{P}}_{{\text{NH}}_{3}}-{\text{P}}_{\text{eq}}}{{\text{P}}_{\text{eq}}}-{\text{k}}_{\text{des}}\text{x}$$

In Equation 3, x is the conversion, k_ads_ and k_des_ are the adsorption and desorption rate constants, respectively. According to the suggested reaction rate expression, when the ammonia pressure is higher than the equilibrium pressure, the adsorption reaction dominates the simultaneous desorption reaction. The degree of the desorption reaction is based on the occupied active site by ammonia and the temperature. When the reaction rate constants are written in terms of Arrhenius law, Equations 5 and 4 can be obtained.


4 $${\text{k}}_{\text{ads}}={\text{As}}_{\text{a}}\text{exp }\left(\frac{-{\text{Ea}}_{\text{a}}}{\text{RT}}\right)$$

5 $${\text{k}}_{\text{des}}={\text{Aa}}_{\text{d}}\text{exp }\left(\frac{-{\text{Ea}}_{\text{d}}}{\text{RT}}\right)$$

Where Aa_a_ and Aa_d_ are preexponential factors and Ea_a_ and Ea_d_ are activation energies for adsorption and desorption reactions, respectively. Adsorption term activation energy and preexponential factors are directly taken from the literature [4] and calculated at different temperatures. With the help of the thermodynamic relation between the equilibrium constant and the standard Gibbs energy change of reaction, the equilibrium constants at those temperatures are also calculated. Since the ratio of k_ads_ to k_des_ gives the equilibrium constant, k_des_ values are calculated at those temperatures. Then, by Arrhenius law, the desorption activation energy and preexponential factor are calculated and tabulated in Table 1 which are used for the suggested rate expression given in Equation 3.

### 2.3. The geometry and description of the reactive system

**Figure 1:**
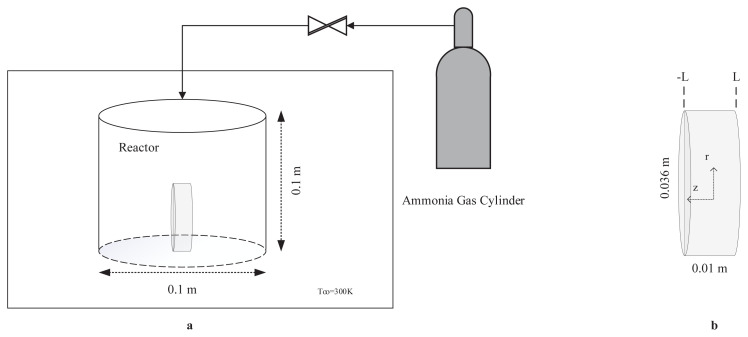
a) Schematic representation of model geometry b) Detailed illustration of solid salt pellet.

The pellet disc of MgCl_2_ grains is placed in a cylinder reactor with 0.1 m diameter and 0.1 m length. The initial radius and length of the pellet are 1.8 × 10^−2^ m and 1 × 10^−2^ m, respectively. The reactor is in a constant temperature sink which is 300 K. Initially the system is under vacuum, hence there is no ammonia. Gaseous ammonia is introduced to the reactor at t = 0 at a pressure higher than the equilibrium pressure corresponding to 300 K, and it starts to diffuse through the pellet. While MgCl_2_ and ammonia react according to Equation 3, the amount of the gaseous ammonia inside the reactor is depleted. At the same time, the volume of the pellet increases because of the reaction, and therefore the available volume of ammonia inside the reactor decreases which affects the pressure of ammonia. Therefore, the bulk ammonia pressure changes with respect to time due to diffusion, reaction, and decrease in the free reactor volume. Since the amount of the adsorbed ammonia is proportional to the used amount of the salt, the reactor volume is selected based on the amount of the salt so that appreciable pressure change occurs in the reactor during the adsorption reaction. Moreover, the temperature of the reactive salt pellet first increases because of the exothermic nature of the adsorption reaction. As the reaction nears the equilibrium, the pellet cools down and its temperature eventually drops to the surrounding temperature. The porosity of the pellet is assumed to be constant in spite of the pellet volume increases due to the reaction. The schematic representation of the model geometry is given in Figure 1.

### 2.4. Modelling heat and mass transfer

In the mathematical model, the following simplifying assumptions are made:

➢ Grains are spherical and have uniform sizes,
➢ The number of grains in the pellet remains constant,
➢ The external mass transfer resistance between the pellet surface and NH_3_ gas in the reactor is negligible,
➢ The porosity in the pellet remains constant in spite of the increase in the grain size during the adsorption reaction.

This assumption is considered reasonable since the pellet is not confined and is free to expand,

➢ Diffusion is the mass transfer mechanism of NH_3_ in the pellet. It is unidirectional, i.e. the axial direction only,
➢ Heat capacity and thermal conductivity of ammonia and metal salt are constant,
➢ Temperature of in the reactor remains constant at the sink temperature.

Ammonia gas in the reactor follows the ideal gas equation of state. The energy transport equation can be written as follows:


6 $$\frac{\partial \text{T}}{\partial \text{z}}\left[{\text{k}}_{\text{eff}}\frac{\partial \text{T}}{\partial \text{z}}\right]+{\text{S}}_{0}\Delta {\text{H}}_{\text{rxn}}=\rho_{\text{p}}\cdot C_{\text{p},\text{p}}\cdot \frac{\partial \text{T}}{\partial \text{t}}$$

In equation 6, k_eff_ (W m^−1^ K^−1^) is the pellet’s effective thermal conductivity, ρ_p_ (kg m^−3^) is the pellet density, C_p,p_ (J kg^−1^ K^−1^) is the pellet heat capacity and *S*_0_ is the source term due to the chemical reaction and given in Equation 7.


6 $$\frac{\partial \text{T}}{\partial \text{z}}\left[{\text{k}}_{\text{eff}}\frac{\partial \text{T}}{\partial \text{z}}\right]+{\text{S}}_{0}\Delta {\text{H}}_{\text{rxn}}=\rho_{\text{p}}\cdot C_{\text{p},\text{p}}\cdot \frac{\partial \text{T}}{\partial \text{t}}$$

7 $${\text{S}}_{0}=\delta {\text{N}}_{\text{salt}}\frac{\text{dx}}{\text{dt}}$$

where δ is the stoichiometric coefficient and N_salt_ is the number of moles per unit volume of the reactive medium. Because of the exothermic nature of the adsorption reaction, heat is generated. The pellet density, effective thermal conductivity, and effective heat capacity, respectively, can be obtained from:


8 $$\rho_{\text{p}}=\rho_{\text{reactant}}\cdot \frac{{\text{V}}_{\text{reactant}}}{{\text{V}}_{\text{total},\text{solid}}}+\rho_{\text{product}}\cdot \frac{{\text{V}}_{\text{product}}}{{\text{V}}_{\text{total},\text{solid}}}+\rho_{{\text{NH}}_{3}}\cdot ɛ$$

9 $${\text{k}}_{\text{eff}}={\text{k}}_{\text{reactant}}\cdot \frac{{\text{V}}_{\text{reactant}}}{{\text{V}}_{\text{total},\text{solid}}}+{\text{k}}_{\text{product}}\cdot \frac{{\text{V}}_{\text{product}}}{{\text{V}}_{\text{total},\text{solid}}}+{\text{k}}_{{\text{NH}}_{3}}\cdot ɛ$$

10 $${\text{c}}_{\text{p},\text{p}}={\text{c}}_{\text{p},\text{reactant}}\cdot \frac{{\text{V}}_{\text{reactant}}}{{\text{V}}_{\text{total},\text{solid}}}+{\text{c}}_{\text{p},\text{product}}\cdot \frac{{\text{V}}_{\text{product}}}{{\text{V}}_{\text{total},\text{solid}}}+{\text{c}}_{\text{p},{\text{NH}}_{3}}\cdot ɛ$$

The initial and boundary conditions are:


11 $$\text{at z}=0\;\;\;\frac{\partial \text{T}}{\partial \text{z}}=0\;(\text{Symmetric boundary condition at the center of the pellet})$$

12 $$\text{at z}=\text{L }\;\;-{\text{k}}_{\text{eff}}\frac{\partial \text{T}}{\partial \text{z}}={\text{h}}_{{\text{NH}}_{3}}\;(\text{T}-{\text{T}}_{\infty })(\text{Heat flux at the surfacr of the pellet})$$

13 at t=0         T=300 K(Uniform temperature inside the pellet)

h_NH3_ (W m^−2^ K^−1^) is the convective heat transfer coefficient and T_∞_ is the temperature of the sink, e.g., a constant temperature water bath. The convective heat transfer coefficient is evaluated by means of the Churchill-Chu correlation.

The governing mass transport equation is given by:


14 $$\frac{\partial }{\partial \text{z}}\left({\text{D}}_{\text{eff}}\frac{\partial }{\partial \text{z}}\left(\frac{{\text{P}}_{{\text{NH}}_{\text{s}}}}{\text{RT}}\right)\right)-{\text{S}}_{0}=\frac{\partial }{\partial \text{t}}\left(\frac{ɛ{\text{P}}_{{\text{NH}}_{\text{s}}}}{\text{RT}}\right)$$

In Equation 14, P_NH3_ is the pointwise pressure of NH_3_ gas in the pellet, ɛ is the porosity of the pellet. D_eff_ (m^2^ s^−1^) is the effective diffusion coefficient of NH_3_ in the pellet. Its temperature dependency is represented via


15 $$\frac{{\text{D}}_{\text{eff}}}{{\text{D}}_{0}}={\left(\frac{\text{T}}{{\text{T}}_{0}}\right)}^{3/2}$$

The following boundary and initial conditions are used:


16 $$\text{at z}=0\;\;\;\frac{\partial \text{P}}{\partial \text{z}}=0\;(\text{Symmetric boundary condition at the center of the pellet})$$

17 $$\text{at z}=\text{L }\;\;{\text{P}}_{{\text{NH}}_{3}}={\text{P}}_{\text{bulk}}\;(\text{Dirichlet boundary condition})$$

18 $$\text{at t}=0\;\;\;{\text{P}}_{{\text{NH}}_{3}}=0\;(\text{Uniform pressure inside the pellet})$$

P_bulk_ is the time-dependent ammonia pressure in the reactor, which decreases as the reaction progresses. The parameters used in the model are tabulated in Table 2.

### 2.5. Numerical methodology

The explicit finite difference method is used for the numerical analysis of the differential equation set in Equations 6 – 18. The pellet is divided into N number of discrete points in the axial direction. In the simulations, axisymmetry is considered. Therefore; initially each control volume thickness, Δz, is L/(N-1), where L is half of the initial total thickness of the slab pellet. N is set as 10. As the reaction proceeds at different extend with respect to axial position, the increase in the thicknesses of the control volumes also becomes position dependent. In order to convert the continuous form of the governing differential equations and the initial and boundary conditions forward in time and centered in space, a finite difference scheme is adopted. Stability is provided if 0≤γ≤0.5 where γ=D_eff_ Δt/(Δz)^2^ for the diffusion equation and γ=k_eff_ Δt/(Δz)^2^ for the heat equation. For accuracy, γ is selected as 0.02, and integration time is chosen as the minimum of γ=D_eff_ Δt/(Δz)^2^ and γ=k_eff_ Δt/(Δz)^2^.

## 3. Results and discussion

**Figure 2:**
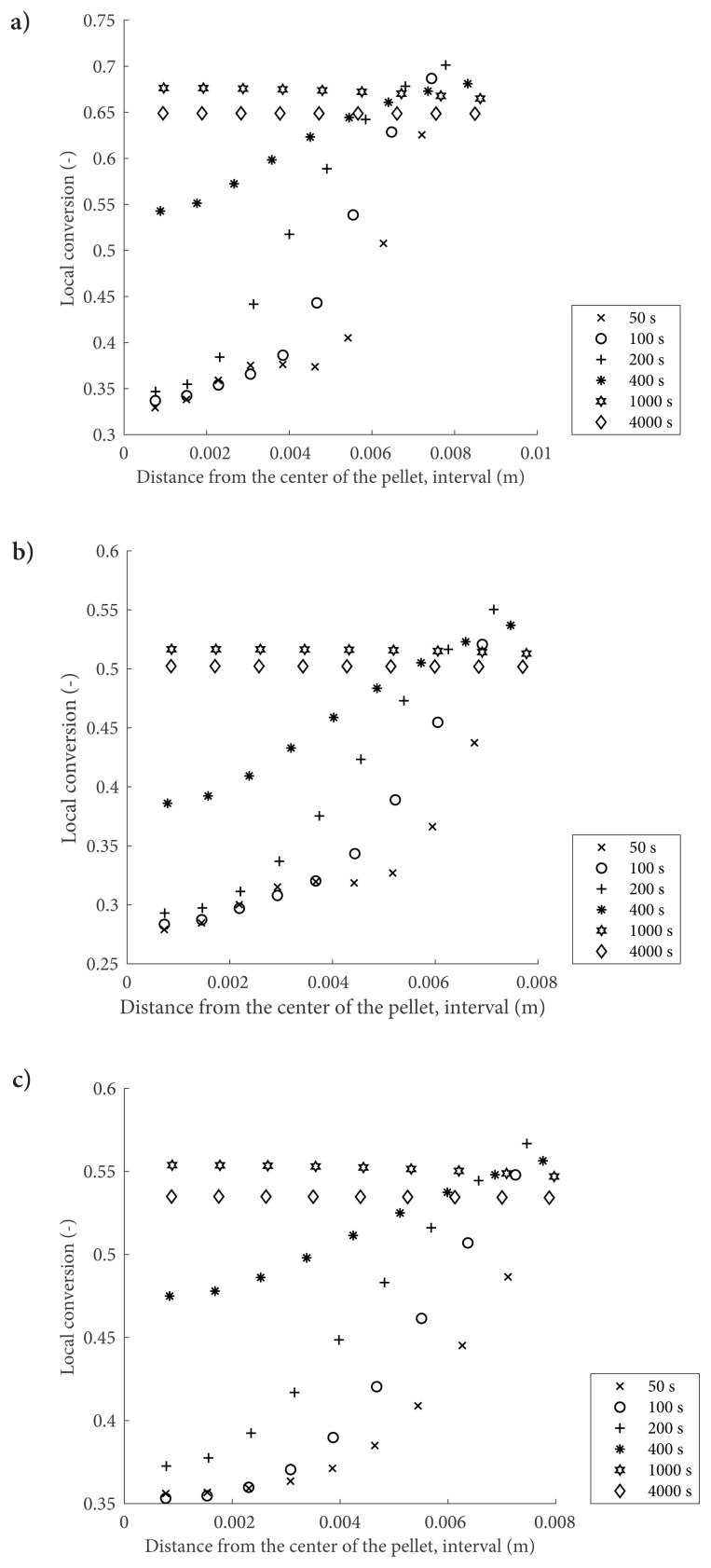
Local conversions for MgCl_2_–NH_3_ reactive system when the operating temperature is 300 K a) when ɛ = 0.2, P initial = 10 bar b) when ɛ = 0.2, P initial = 5 bar and c) when ɛ = 0.3, P initial = 10 bar.

Figure 2 exhibits the impact of the pellet porosity and initial reactor NH_3_ pressure on the transient local conversion profiles at the operating temperature of 300 K. In all three cases, at earlier times, local conversions closer to the pellet surface are higher than those closer to the center. As the reaction proceeds, the local conversions increase as expected. In any case, the differences between the surface and center conversions become less pronounced compared to the earlier profiles. This characteristic of the salt conversion profiles stems from the combined effect of the coupled heat and mass transfer and volume increase on the local reaction rates. Comparison between Figures 2(a) and 2(b) indicates the effect of initial NH_3_ pressure hence concentration in the reactor. With the increased pressure, the reaction rate becomes higher, as suggested by the faster evolution of conversion profiles at 10 bars compared to the 5 bars case. Another interesting feature is that, increased availability of NH_3_ at higher pressure leads to a higher salt conversion and volume increase. There are two main effects of the pellet porosity on the conversion profiles which are depicted in Figures 2(a) and 2(c). Lower mass transfer resistance associated with increased porosity is revealed through the faster evolution of the earlier profiles in Figure 2(c) than those in Figure 2(a). Higher porosity at a fixed pellet volume implies a lower amount of salt available, hence a lower potential of volume increases due to the reaction. Increasing porosity from 0.2 to 0.3 clearly resulted in a lower conversion and lower volume increase, as shown in Figures 2 (a) and 2(c) and Figure 5. The local salt temperature has a higher value than the bulk temperature (300 K) at 1000 s, which enhances the desorption reaction. Hence, in all cases, the equilibrium conversion is slightly lower than the maximum conversion because of the reversible nature of the system.

**Figure 3:**
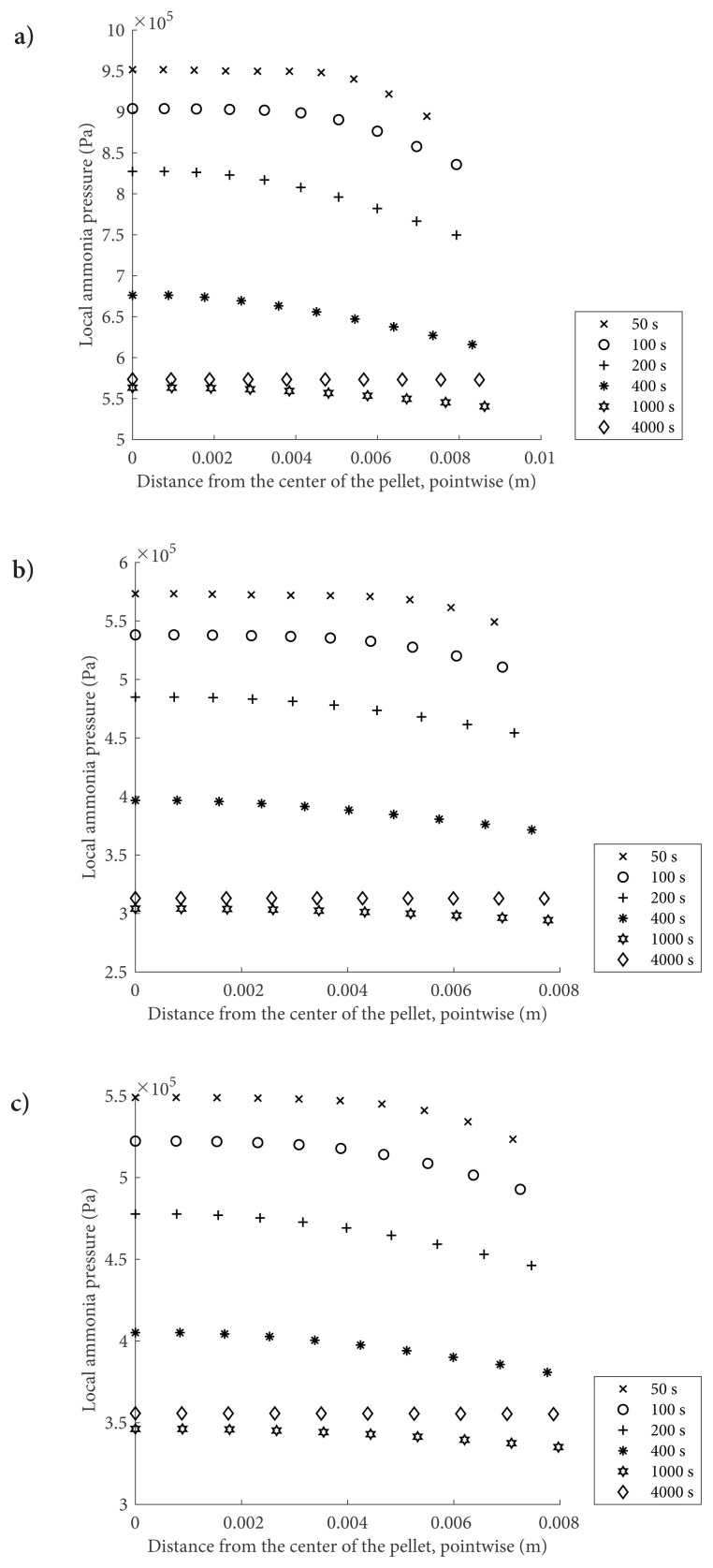
Local pressure values for MgCl_2_–NH_3_reactive system when the operating temperature is 300 K a) when ɛ = 0.2, P initial = 10 bar b) when ɛ = 0.2, P initial = 5 bar and c) when ɛ = 0.3, P initial = 10 bar.

In Figure 3, local ammonia pressure profiles are given. It is clearly seen that the pressure gradient is not significant in the solid pellet. Equation 3 indicates that the deviation between the operating pressure and equilibrium pressure is the driving force for the adsorption, which is the reason for the high adsorption rate at the start. Therefore, as the NH_3_ is depleted or as the reactor pressure gets lower during the process, the rate of conversion becomes slower and slower. The dependence of reaction rate on pressure is also revealed through the comparison between Figures 3 (a) and 3(b), where the operating pressures are 10 bars and 5 bars, respectively. Since the initial temperature is the same as 300 K, the initial equilibrium pressures are identical for all cases. In the beginning, because of the high initial equilibrium drop, the reaction rate is high in the case of 10 bars. Due to the higher initial conversion, pellet volume increase has the highest value for this case. This increase leads to additional diffusion resistance; hence, the initial pressure gradient is more dominant in this case. The porosity effect is observed in Figures 3 (a) and 3(c). More salt amount in the 0.2 porosity case leads to a higher temperature increase at the early stages of the reaction. This temperature increase enhances the impact of the desorption reaction. Therefore, it leads to a decrease in the pressure drop rate, as seen in the same figures.

**Figure 4:**
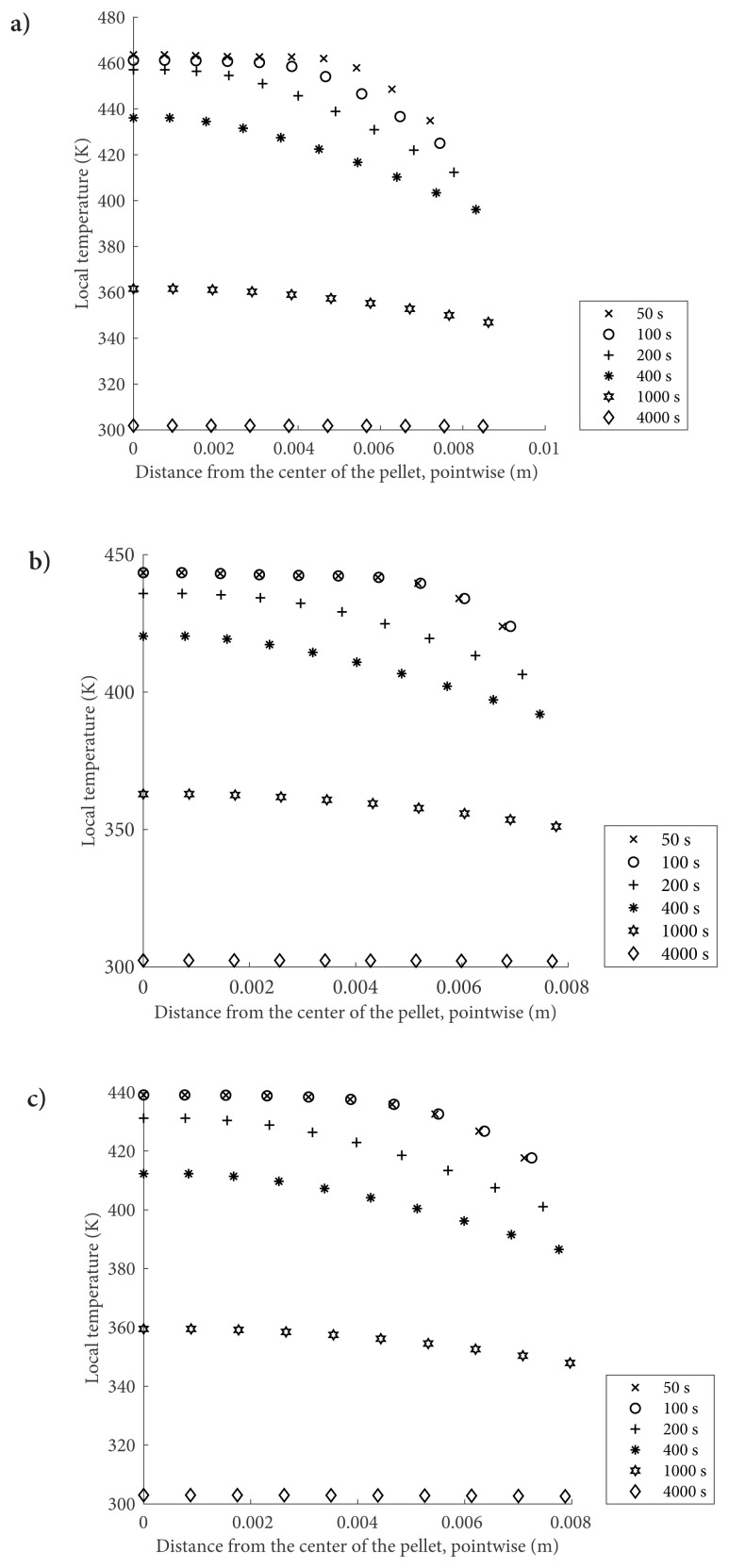
Local temperature values for MgCl_2_–NH_3_reactive system when the operating temperature is 300 K a) when ɛ = 0.2, P initial = 10 bar b) when ɛ = 0.2, P initial = 5 bar and c) when ɛ = 0.3, P initial = 10 bar.

Transient temperature distributions for the investigated three cases are depicted in Figure 4. The heat transfer characteristics of the three cases exhibit similar behavior both quantitatively and qualitatively, despite the porosity and reactor pressure variations. Due to the exothermic adsorption reaction, pellet temperature in each case increases quickly. The cooling occurs from the pellet surfaces to the reactor gas that is kept at 300 K. Therefore, lower temperatures at the locations closer to the surface compared to those closer to the pellet center are observed. These strong temperature gradients, especially at the earlier times of the operation, indicate strong heat transfer resistance associated with the poor thermal conductivity of the metal salt. The local temperature increase is also higher for 10 bars case because of the heat of the adsorption reaction. The model captures the change of equilibrium pressure with respect to the local temperature, which considerably affects conversion calculation. However, when the highest local temperature values are considered for the case of 10 bars and 5 bars, the equilibrium drop is still higher in the 10 bars case, leading to a higher reaction rate than that of the 5 bars case.

In CHP applications, adsorbent material should have very low thermal resistance to ensure quick cooling and heating of the adsorbate material during the adsorption and desorption cycles, respectively. The heat transfer restriction should be handled to benefit from the high NH_3_ adsorption capacities of earth-alkaline metal salts as CHP adsorbent material. For example, the geometry of the bed can be designed to shrink the distances for heat transfer so that quick heating and cooling can be provided. Local conversion profiles and local temperature profiles in Figure 2 and Figure 4 agree with each other well. Since adsorption is favored at low temperatures at a given time, local conversions are higher at the lower temperature locations closer to the surface. Hence, another benefit of low thermal conductivity would be the effective utilization of the pellet in terms of uniform adsorption rates.

**Figure 5:**
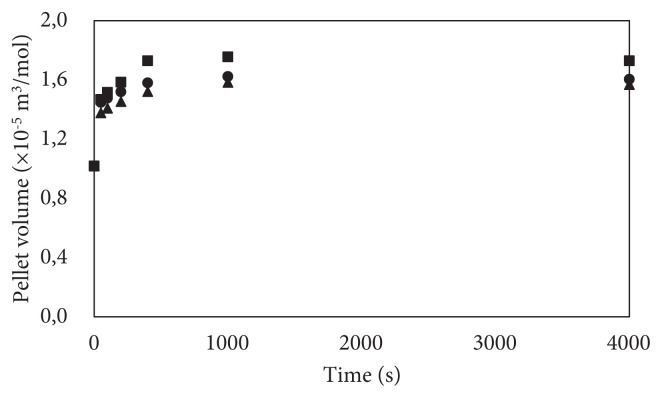
Pellet volume increase with reaction progress: P initial = 10 bar and ɛ = 0.2, square dots, P initial = 10 bar and ɛ = 0.3, circle dots, P initial = 5 bar and ɛ = 0.2, triangle dots.

The pellet volume increase with respect to time is given in Figure 5. The porosity is assumed to remain constant. In all cases, appreciable volume changes are observed. The highest volume increase occurs when the porosity is 0.2, and the initial NH_3_ pressure in the reactor is 10 bars, which is expected because volume change is directly proportional to the available materials. Hence, the lower pressure of 5 bars leads to the lowest volume change. In practical applications, this irreversible volume change should be considered to keep the structural integrity of the reactor. At the equilibrium, conversion is lower than that of maximum conversion because of the desorption reaction and pellet volume also decreases slightly at equilibrium. The studies express that pellet volume increases during the adsorption reaction while the pellet does not shrink during the desorption reaction [6]. Therefore, the model simulation needs further improvement based on the increase in pellet volume.

**Figure 6:**
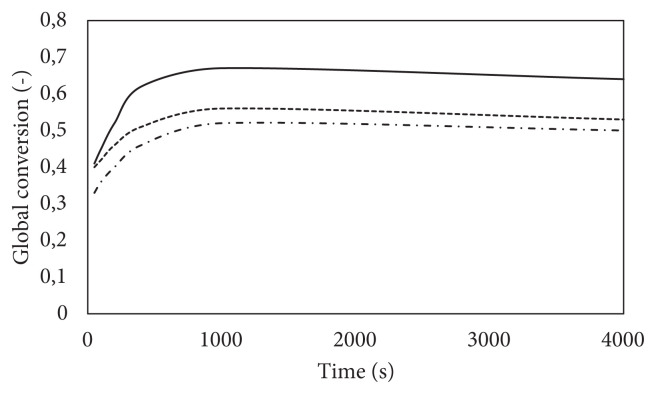
Global conversion with respect to time: P initial = 10 bar and ɛ = 0.2, solid line, P initial = 10 bar and ɛ = 0.3, square dot line, P initial = 5 bar and ɛ = 0.2, dash-dot line.

Global conversion is calculated by the ratio of product amount to the total salt amount, and it is represented in Figure 6. As indicated previously, the highest conversion is observed in the case of 10 bar pressure and 0.2 porosity due to the highest available reactant amounts. The model results were compared with the study of Iwata et al. in which MgCl_2_ salt was mixed with carbon fiber to enhance thermal conductivity [4]. The reactor was connected to a large gas cylinder to minimize the pressure change. For the comparison, the reactor length and diameter are each defined as 10 m. The model is compared for the case of 4 bars and 423 K. In the Iwata et al. study, the global conversion reached almost 1 in 50 s, while in this model, only 0.7 conversion is achieved in 200 s. One of the reasons may be that the mixture of salt and carbon fiber is put into the reactor by hand press in the Iwata et al. study, which leads to a higher porosity than our model that advances mass transport. Another important aspect is that using carbon fiber increased thermal conductivity up to 3 W m^−1^ K^−1^ while in the presented model, this property is around 0.4 W m^−1^ K^−1^. Increasing the thermal conductivity of the pellet leads to enhanced thermal diffusion, which is the main limitation. At the beginning of the adsorption reaction temperature of the salt suddenly increases, and almost in 7500 s, it decreases to the bulk temperature. An increase in temperature promotes the desorption reaction and hence conversion increase is not high such as given in the Iwata’s study. Still, the reaction rate kinetics needs further experimental research.

## 4. Conclusions

The results of this study allow one to draw the following conclusions:

- Due to the strong interaction between heat and mass transfer and reaction kinetics, local conversions in the pellet differ appreciably within the pellet suggesting inefficient use of the pellet.
- Almost uniform NH_3_ concentrations in the pellet indicate negligible mass transfer resistance.
- An increase in the operating pressure leads to a higher reaction rate because of the deviation from the equilibrium.
- Due to the low thermal conductivity of the pellet, there is a strong temperature gradient, especially at the earlier times of the reaction.

## Figures and Tables

**Table 1:** Adsorption term parameters of the reaction obtained from reference [6] and calculated desorption term parameters.

Kinetic parameter	Physical meaning	Value
Aa_a_ (s^−1^)	Adsorption preexponential factor	4.31×10^6^
Ea_a_ (J/mol)	Adsorption activation energy	67,000
Aa_d_ (s^−1^)	Desorption preexponential factor	2.76
Ea_d_ (J/mol)	Desorption activation energy	20,277

**Table 2:** Material properties of MgCl_2_.2NH_3_ and MgCl_2_.6NH_3_ [6].

Physico-chemical property	MgCl_2_.2NH_3_	MgCl_2_.6NH_3_
ρ (kg m^−3^)	1703	1252
Cp (J kg^−1^ K^−1^)	1210	1600
Vm (m^3^ mol^−1^)	7.59 × 10^−5^	1.57 × 10^−4^
Mw (g mol^−1^)	129.3	197.4
